# Comparative Analysis of the Effects of Hydroxysafflor Yellow A and Anhydrosafflor Yellow B in Safflower Series of Herb Pairs Using Prep-HPLC and a Selective Knock-Out Approach

**DOI:** 10.3390/molecules21111480

**Published:** 2016-11-06

**Authors:** Cheng Qu, Lin-Yan Wang, Wen-Tao Jin, Yu-Ping Tang, Yi Jin, Xu-Qin Shi, Li-Li Shang, Er-Xin Shang, Jin-Ao Duan

**Affiliations:** 1 Jiangsu Collaborative Innovation Center of Chinese Medicinal Resources Industrialization, and National and Local Collaborative Engineering Center of Chinese Medicinal Resources Industrialization and Formulae Innovative Medicine, and Jiangsu Key Laboratory for High Technology Research of TCM Formulae, Nanjing University of Chinese Medicine, Nanjing 210023, China; qucheng9527@163.com (C.Q.); linyanwang1990@126.com (L.-Y.W.); yijin2020@126.com (Y.J.); shixuqin@126.com (X.-Q.S.); shanglilicpu@163.com (L.-L.S.); shex@njucm.edu.cn (E.-X.S.); dja@njucm.edu.cn (J.-A.D.); 2 No. 29 High School, Nanjing 210036, China; jwtnj@126.com

**Keywords:** selective knock-out approach, safflower, *Carthamus tinctorius*, herb pair, hydroxysafflor yellow A, anhydrosafflor yellow B

## Abstract

The flower of *Carthamus tinctorius* L. (Carthami Flos, safflower), important in traditional Chinese medicine (TCM), is known for treating blood stasis, coronary heart disease, hypertension, and cerebrovascular disease in clinical and experimental studies. It is widely accepted that hydroxysafflor yellow A (HSYA) and anhydrosafflor yellow B (ASYB) are the major bioactive components of many formulae comprised of safflower. In this study, selective knock-out of target components such as HSYA and ASYB by using preparative high performance liquid chromatography (prep-HPLC) followed by antiplatelet and anticoagulation activities evaluation was used to investigate the roles of bioactive ingredients in safflower series of herb pairs. The results showed that both HSYA and ASYB not only played a direct role in activating blood circulation, but also indirectly made a contribution to the total bioactivity of safflower series of herb pairs. The degree of contribution of HSYA in the safflower and its series herb pairs was as follows: Carthami Flos-Ginseng Radix et Rhizoma Rubra (CF-GR) > Carthami Flos-Sappan Lignum (CF-SL) > Carthami Flos-Angelicae Sinensis Radix (CF-AS) > Carthami Flos-Astragali Radix (CF-AR) > Carthami Flos-Angelicae Sinensis Radix (CF-AS) > Carthami Flos-Glycyrrhizae Radix et Rhizoma (CF-GL) > Carthami Flos-Salviae Miltiorrhizae Radix et Rhizoma (CF-SM) > Carthami Flos (CF), and the contribution degree of ASYB in the safflower and its series herb pairs: CF-GL > CF-PS > CF-AS > CF-SL > CF-SM > CF-AR > CF-GR > CF. So, this study provided a significant and effective approach to elucidate the contribution of different herbal components to the bioactivity of the herb pair, and clarification of the variation of herb-pair compatibilities. In addition, this study provides guidance for investigating the relationship between herbal compounds and the bioactivities of herb pairs. It also provides a scientific basis for reasonable clinical applications and new drug development on the basis of the safflower series of herb pairs.

## 1. Introduction

Chinese herbal medicine, with a history of thousands of years, is one of the oldest traditional medicines in the world and has definite clinical effect, especially in the treatment of chronic diseases. Its worldwide practice has recently increased in both developing and developed countries [1,2,3]. With the accumulation of the therapeutic experience, many traditional Chinese medicine (TCM) practitioners realized that the pathogenesis and progression of diseases are complicated and the effect of a single drug may be modest and/or hampered by various side effects or resistance in the clinic [4]. So, a combination of two or more herbs could not only be much more effective, but could also reflect some interesting interactions between herbs; thus, formulae have since been gradually developed, and have shown significance in long-term clinical practice [5]. It is universally acknowledged that the same medicine may form different formulae according to the monarch, minister, assistant, and guide theory; the medicine and its active ingredients may play different roles in the different formulae [6]. Herb pairings, unique combinations of two relatively fixed herbs in the clinic, is the most fundamental and the simplest form of multi-herb therapy to achieve specific efficacy by a peculiar methodology [7,8]. So, an herb may play different roles in different herb pairs. Without altering the basic therapeutic features of multi-herb formulae, herb pairs retain the fundamental composition characteristic of Chinese herbal formulae and have gradually become one of the focuses of modern TCM research [9].

**Figure 1:**
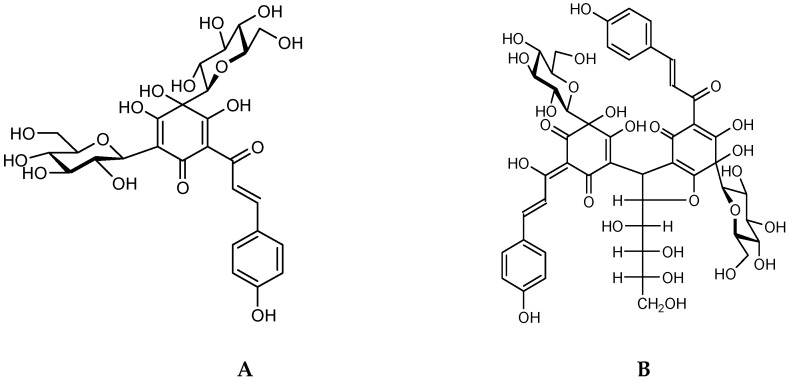
Chemical structures of HSYA (**A**) and ASYB (**B**).

The florets of *Carthamus tinctorius* L. (Carthami Flos, CF), which is a common TCM used in China, Japan, Korea, and other Asian countries, are known as safflower [10]. Modern pharmacological experiments have demonstrated that safflower possesses wide-reaching biological effects, such as dilating the coronary artery, improving myocardial ischemia, anticoagulation, antithrombosis, anti-oxidation, etc. [11]. Safflower contains several bioactive constituents, but the quinochalcone *C*-glycosides are considered its characteristic and bioactive compounds. Meanwhile, hydroxysafflor yellow A (HSYA) and anhydrosafflor yellow B (ASYB) (Figure 1), as the most important bioactive quinochalcone *C*-glycosides, play a significant role in promoting blood circulation and removing blood stasis in safflower. According to TCM theory, safflower is often combined with other TCMs to treat related diseases of blood stasis syndrome clinically, such as Carthami Flos-Angelicae Sinensis Radix (CF-AS), Carthami Flos-Astragali Radix (CF-AR), and Carthami Flos-Salviae Miltiorrhizae Radix et Rhizoma (CF-SM) as herb pairs [12]. However, few related studies were reported about the role of safflower and its bioactive components in these different herb pairs.

In our previous studies, we have established a convenient and effective method for the comprehensive application of a selective knock-out approach with preparative high-performance liquid chromatography (prep-HPLC) technology and biological activity evaluation to investigate the contribution of paeoniflorin and senkyunolide I in SiWu decoction. The results showed that both paeoniflorin and senkyunolide I not only directly brought about some bio-activities, but also indirectly made a contribution to the total bio-activity reflection of SiWu decoction [13]. Drawing on the method, we attempted to knock out HSYA and ASYB from safflower series of herb pairs respectively by prep-HPLC, then comparative bioactivity evaluation was used to investigate the effect of HSYA and ASYB in these herb pairs. In the study, we chose eight classic safflower series of herb pairs in the clinical remedies for activating blood circulation including CF and Persicae Semen (PS), SM, AS, Sappan Lignum (SL), AR, Ginseng Radix et Rhizoma Rubra (GR), and Glycyrrhizae Radix et Rhizoma (GL), and then antiplatelet and anticoagulation activity assays were applied to comparatively evaluate their bioactivities.

## 2. Results and Discussion

### 2.1. The Knock-Out Results of Preparative HPLC

**Figure 2:**
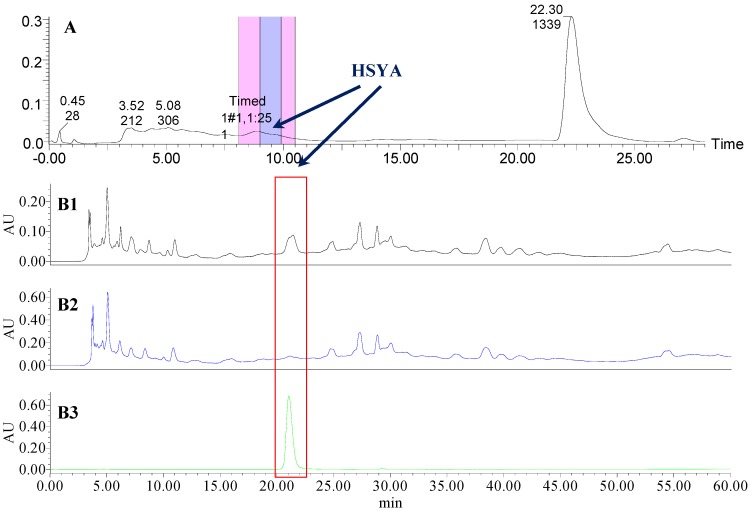
The preparative and analytical chromatograms of the depletion of HSYA from CF; (**A**) preparative chromatogram at 270 nm; (**B1**) analytical chromatogram of CF at 270 nm; (**B2**) analytical chromatogram of CF-A at 270 nm; (**B3**) analytical chromatogram of HSYA depleted from CF at 270 nm.

**Figure 3:**
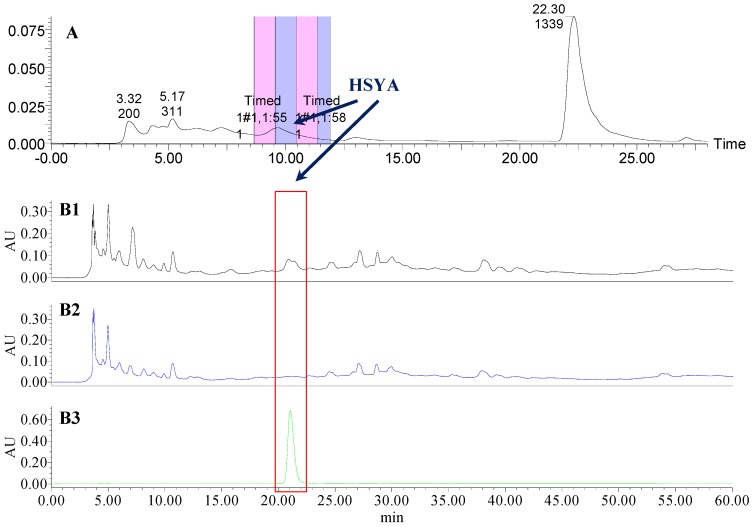
The preparative and analytical chromatograms of the depletion of HSYA from CF-AS; (**A**) preparative chromatogram at 270 nm; (**B1**) analytical chromatogram of CF-AS at 270 nm; (**B2**) analytical chromatogram of CF-AS-A at 270 nm; (**B3**) analytical chromatogram of HSYA depleted from CF-AS at 270 nm.

**Figure 4:**
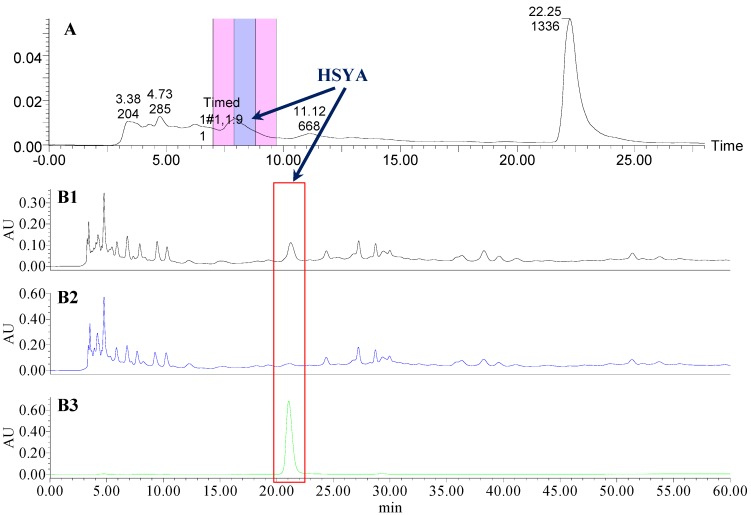
The preparative and analytical chromatograms of the depletion of HSYA from CF-AR; (**A**) preparative chromatogram at 270 nm; (**B1**) analytical chromatogram of CF-AR at 270 nm; (**B2**) analytical chromatogram of CF-AR-A at 270 nm; (**B3**) analytical chromatogram of HSYA depleted from CF-AR at 270 nm.

**Figure 5:**
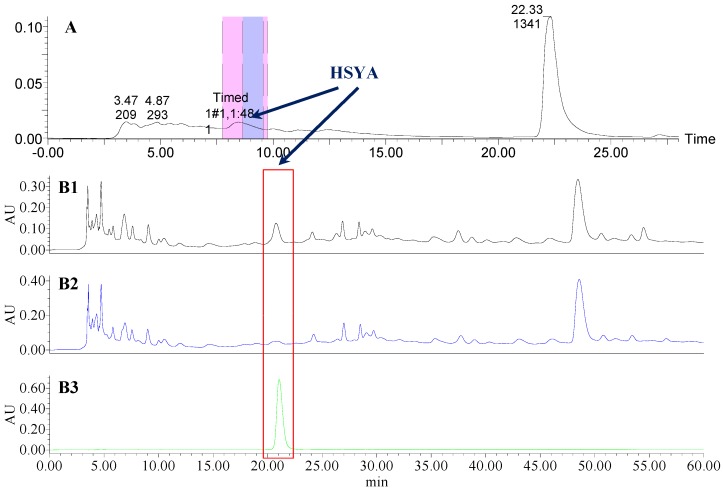
The preparative and analytical chromatograms of the depletion of HSYA from CF-SM. (**A**) preparative chromatogram at 270 nm; (**B1**) analytical chromatogram of CF-SM at 270 nm; (**B2**) analytical chromatogram of CF-SM-A at 270 nm; (**B3**) analytical chromatogram of HSYA depleted from CF-SM at 270 nm.

**Figure 6:**
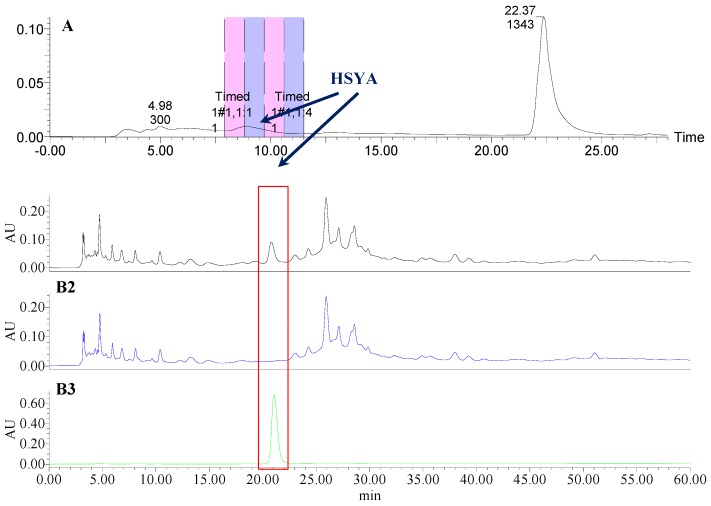
The preparative and analytical chromatograms of the depletion of HSYA from CF-SL; (**A**) preparative chromatogram at 270 nm; (**B1**) analytical chromatogram of CF-SL at 270 nm; (**B2**) analytical chromatogram of CF-SL-A at 270 nm; (**B3**) analytical chromatogram of HSYA depleted from CF-SL at 270 nm.

**Figure 7:**
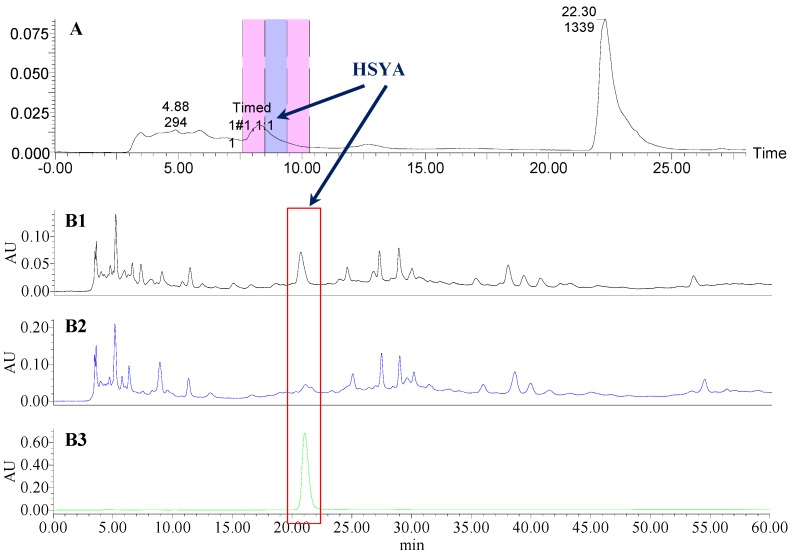
The preparative and analytical chromatograms of the depletion of HSYA from CF-PS; (**A**) preparative chromatogram at 270 nm; (**B1**) analytical chromatogram of CF-PS at 270 nm; (**B2**) analytical chromatogram of CF-PS-A at 270 nm; (**B3**) analytical chromatogram of HSYA depleted from CF-PS at 270 nm.

**Figure 8:**
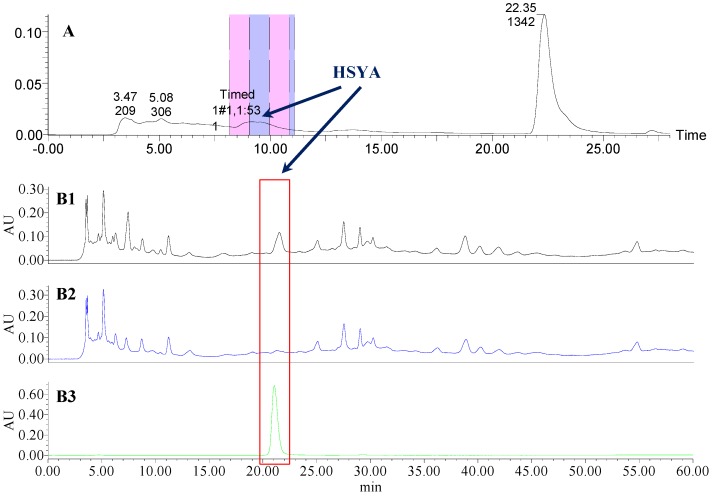
The preparative and analytical chromatograms of the depletion of HSYA from CF-GR; (**A**) preparative chromatogram at 270 nm; (**B1**) analytical chromatogram of CF-GR at 270 nm; (**B2**) analytical chromatogram of CF-GR-A at 270 nm; (**B3**) analytical chromatogram of HSYA depleted from CF-GR at 270 nm.

**Figure 9:**
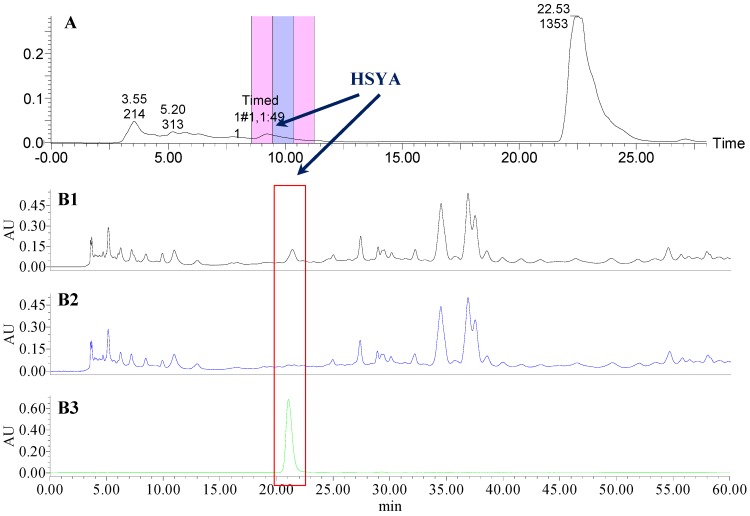
The preparative and analytical chromatograms of the depletion of HSYA from CF-GL. (**A**) preparative chromatogram at 270 nm; (**B1**) analytical chromatogram of CF-GL at 270 nm; (**B2**) analytical chromatogram of CF-GL-A at 270 nm; (**B3**) analytical chromatogram of HSYA depleted from CF-GL at 270 nm.

**Figure 10:**
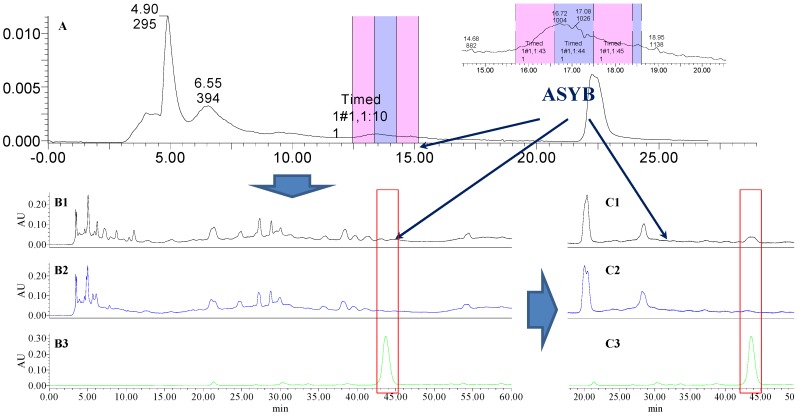
The preparative and analytical chromatograms of the depletion of ASYB from CF. (**A**) preparative chromatogram at 270 nm; (**B1**) analytical chromatogram of CF at 270 nm; (**B2**) analytical chromatogram of CF-B at 270 nm; (**B3**) analytical chromatogram of ASYB depleted from CF at 270 nm. (**C1**) analytical chromatogram of CF at 400 nm; (**C2**) analytical chromatogram of CF-B at 400 nm; (**C3**) analytical chromatogram of ASYB depleted from CF at 400 nm.

**Figure 11:**
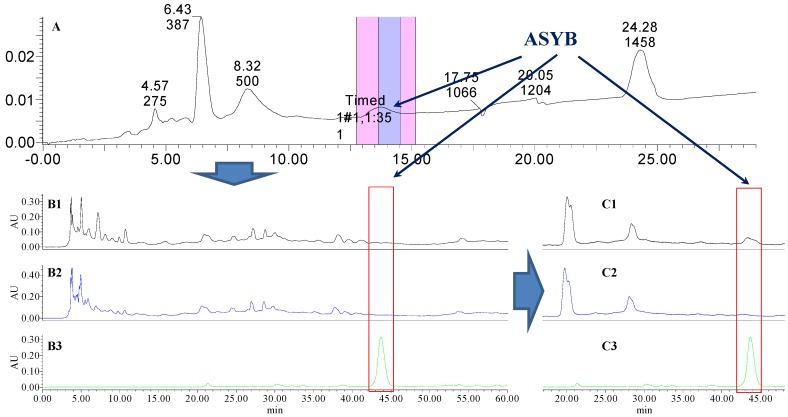
The preparative and analytical chromatograms of the depletion of ASYB from CF-AS. (**A**) preparative chromatogram at 270 nm; (**B1**) analytical chromatogram of CF-AS at 270 nm; (**B2**) analytical chromatogram of CF-AS-B at 270 nm; (**B3**) analytical chromatogram of ASYB depleted from CF-AS at 270 nm. (**C1**) analytical chromatogram of CF-AS at 400 nm; (**C2**) analytical chromatogram of CF-AS-B at 400 nm; (**C3**) analytical chromatogram of ASYB depleted from CF-AS at 400 nm.

**Figure 12:**
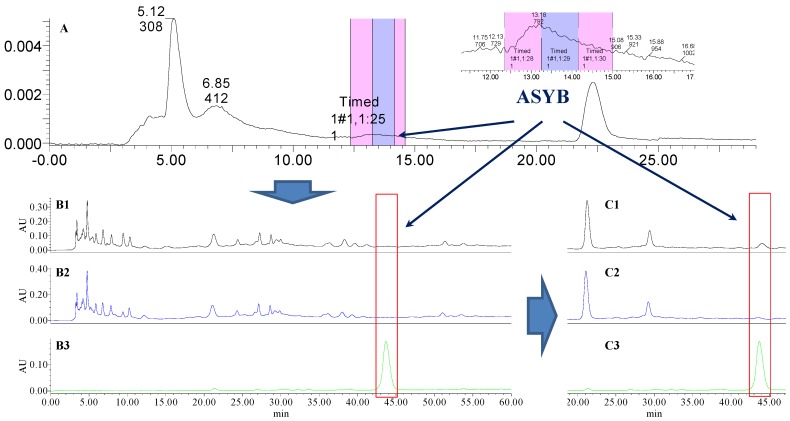
The preparative and analytical chromatograms of the depletion of ASYB from CF-AR. (**A**) preparative chromatogram at 270 nm; (**B1**) analytical chromatogram of CF-AR at 270 nm; (**B2**) analytical chromatogram of CF-AR-B at 270 nm; (**B3**) analytical chromatogram of ASYB depleted from CF-AR at 270 nm. (**C1**) analytical chromatogram of CF-AR at 400 nm; (**C2**) analytical chromatogram of CF-AR-B at 400 nm; (**C3**) analytical chromatogram of ASYB depleted from CF-AR at 400 nm.

**Figure 13:**
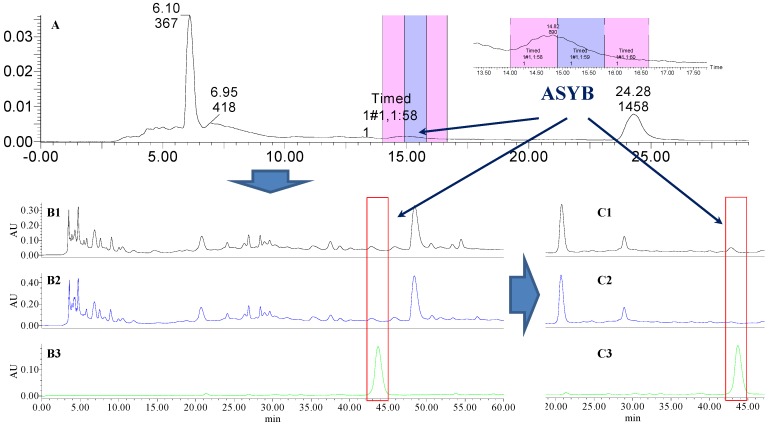
The preparative and analytical chromatograms of the depletion of ASYB from CF-SM. (**A**) preparative chromatogram at 270 nm; (**B1**) analytical chromatogram of CF-SM at 270 nm; (**B2**) analytical chromatogram of CF-SM-B at 270 nm; (**B3**) analytical chromatogram of ASYB depleted from CF-SM at 270 nm. (**C1**) analytical chromatogram of CF-SM at 400 nm; (**C2**) analytical chromatogram of CF-SM-B at 400 nm; (**C3**) analytical chromatogram of ASYB depleted from CF-SM at 400 nm.

**Figure 14:**
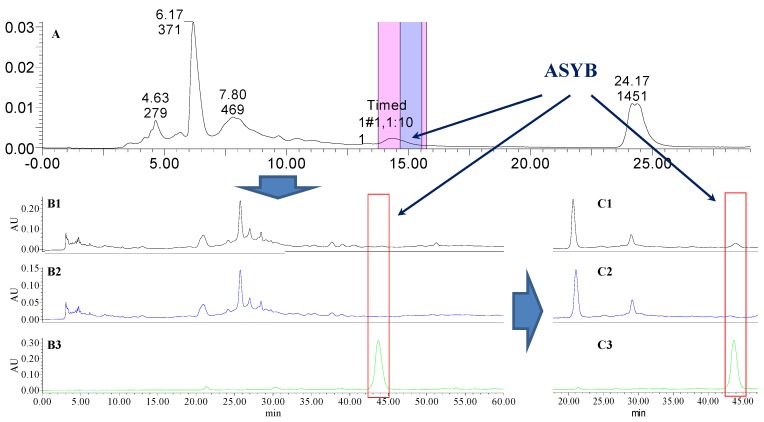
The preparative and analytical chromatograms of the depletion of ASYB from CF-SL. (**A**) preparative chromatogram at 270 nm; (**B1**) analytical chromatogram of CF-SL at 270 nm; (**B2**) analytical chromatogram of CF-SL-B at 270 nm; (**B3**) analytical chromatogram of ASYB depleted from CF-SL at 270 nm. (**C1**) analytical chromatogram of CF-SL at 400 nm; (**C2**) analytical chromatogram of CF-SL-B at 400 nm; (**C3**) analytical chromatogram of ASYB depleted from CF-SL at 400 nm.

**Figure 15:**
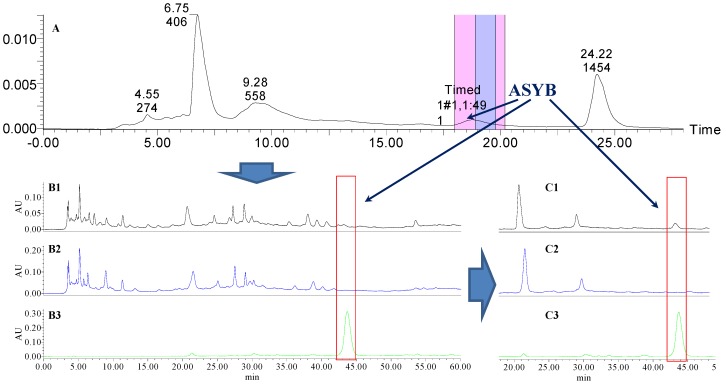
The preparative and analytical chromatograms of the depletion of ASYB from CF-PS. (**A**) preparative chromatogram at 270 nm; (**B1**) analytical chromatogram of CF-PS at 270 nm; (**B2**) analytical chromatogram of CF-PS-B at 270 nm; (**B3**) analytical chromatogram of ASYB depleted from CF-PS at 270 nm. (**C1**) analytical chromatogram of CF-PS at 400 nm; (**C2**) analytical chromatogram of CF-PS-B at 400 nm; (**C3**) analytical chromatogram of ASYB depleted from CF-PS at 400 nm.

**Figure 16:**
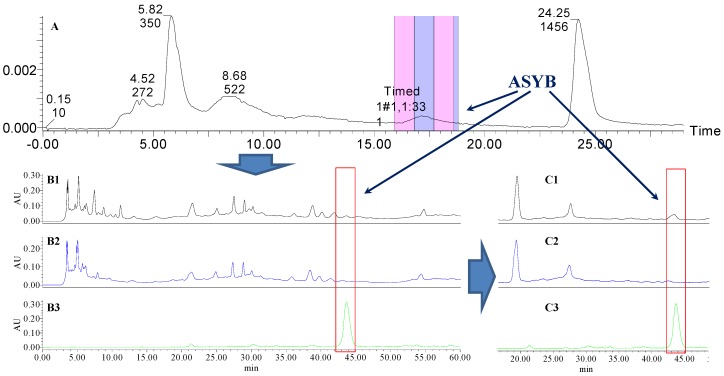
The preparative and analytical chromatograms of the depletion of ASYB from CF-GR. (**A**) preparative chromatogram at 270 nm; (**B1**) analytical chromatogram of CF-GR at 270 nm; (**B2**) analytical chromatogram of CF-GR-B at 270 nm; (**B3**) analytical chromatogram of ASYB depleted from CF-GR at 270 nm. (**C1**) analytical chromatogram of CF-GR at 400 nm; (**C2**) analytical chromatogram of CF-GR-B at 400 nm; (**C3**) analytical chromatogram of ASYB depleted from CF-GR at 400 nm.

**Figure 17:**
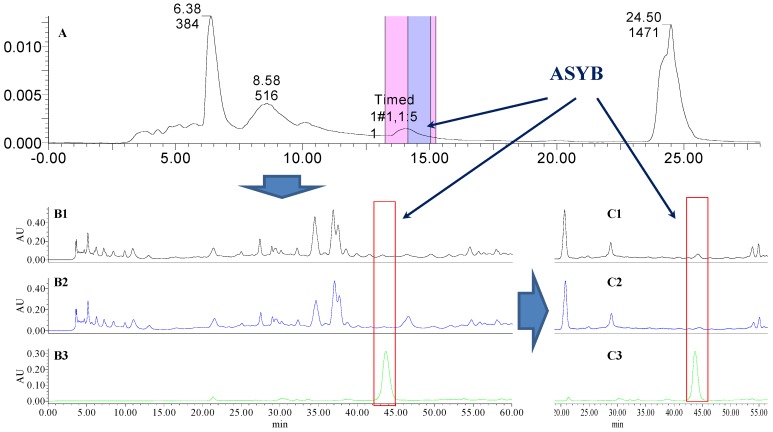
The preparative and analytical chromatograms of the depletion of ASYB from CF-GL. (**A**) preparative chromatogram at 270 nm; (**B1**) analytical chromatogram of CF-GL at 270 nm; (**B2**) analytical chromatogram of CF-GL-B at 270 nm; (**B3**) analytical chromatogram of ASYB depleted from CF-GL at 270 nm. (**C1**) analytical chromatogram of CF-GL at 400 nm; (**C2**) analytical chromatogram of CF-GL-B at 400 nm; (**C3**) analytical chromatogram of ASYB depleted from CF-GL at 400 nm.

Analytical HPLC was used to check the results after knocking out the HSYA or ASYB of safflower series of herb pairs. Through comparing with standard substances and related literature data [14], the two peaks were identified as HSYA (*t_R_* = 21.12 min) and ASYB (*t_R_* = 43.87 min) and with full wavelength scanning analysis by analytical HPLC, ^1^H-NMR and ^13^C-NMR, the results showed that their purity was all more than 85%. In a comparative analysis of the chromatograms of all samples, HSYA and ASYB were successfully knocked out from safflower and its series of herb pairs, respectively, as shown in Figure 2, Figure 3, Figure 4, Figure 5, Figure 6, Figure 7, Figure 8, Figure 9, Figure 10, Figure 11, Figure 12, Figure 13, Figure 14, Figure 15, Figure 16 and Figure 17. Three kinds of samples could be obtained by this method: HSYA and CF-A, CF-AS-A, CF-AR-A, CF-SM-A, CF-SL-A, CF-PS-A, CF-GR-A, CF-GL-A (safflower series of herb pairs knock-out HSYA); ASYB and CF-B, CF-AS-B, CF-AR-B, CF-SM-B, CF-SL-B, CF-PS-B, CF-GR-B, CF-GL-B (safflower series of herb pairs knock-out ASYB). Thereafter, the antiplatelet aggregation and anticoagulation bio-activities were evaluated for all samples.

### 2.2. Effect of PAF-Induced Platelet Aggregation

Platelets play a pivotal role in every phase of atheromatous thrombosis; platelet adhesion, aggregation, and release can all lead to thrombosis [15]. So inhibition of platelet aggregation played an extremely important role in the treatment of cardiovascular diseases. PAF is the strongest platelet aggregation revulsive, so was chosen as the agonist for the in vitro platelet aggregation [16,17]. As shown in Table 1, the potency of inhibition of the PAF-induced platelet aggregation is significantly reduced after knocking out the HSYA with a stock solution in CF, CF-PS, CF-GR, CF-AS, CF-SL, and CF-AR samples; the order of contribution degree is CF-GR > CF-AS > CF > CF-SL > CF-PS > CF-AR. The results demonstrated that HSYA had the greatest effect on PAF-induced platelet aggregation activity in these herb pairs. Yet the role of the ingredient was hidden when CF was combined with GL or SM.

Meanwhile Table 1 shows that the potency of inhibition of PAF-induced platelet aggregation is significantly reduced after knocking out the ASYB in CF, CF-GR, CF-AS, CF-SL samples; the order of contribution degree is CF-SL > CF > CF-GR > CF-AS. Hence ASYB had the greatest effect on PAF-induced platelet aggregation activity in these herb pairs, but the effect of the composition is not obvious when safflower was combined with the other four TCMs.

### 2.3. Effect of AA-Induced Platelet Aggregation

AA is a precursor of TXA_2_ biosynthesis and a potent platelet aggregation promoter and vasoconstrictor of diseases such as thrombosis, cerebral ischemia, shock, et al. for its physiological activities of inducing platelet aggregation and lowering levels of platelet cAMP [18]. Drugs of inhibition of AA metabolism could block the intermediate link of metabolizing TXA_2_ to reduce the generation of TXA_2_ and its damage on vascular endothelial cells. As shown in Table 2, the potency of inhibition of the AA-induced platelet aggregation is significantly reduced after knocking out the HSYA in CF, CF-PS, CF-GL, CF-GR, CF-AS, CF-SL, CF-AR samples; the order of contribution degree is CF-AS > CF-PS > CF-GR > CF-SL > CF-GL > CF-AR > CF. So the results demonstrated that HSYA had the greatest effect on AA-induced platelet aggregation activity in these herb pairs. When CF was combined with SM, the role of the ingredient was hidden.

Table 2 also shows that the potency of inhibition of the AA-induced platelet aggregation is remarkably reduced after depleting the ASYB in CF, CF-PS, CF-GL, CF-GR, CF-AS, and CF-AR samples; the order of contribution degree is CF-AS > CF-PS > CF-GR > CF-GL > CF-AR > CF. Hence ASYB had the greatest effect on AA-induced platelet aggregation activity in these herb pairs, but the effect of the composition is not obvious when safflower was combined with the other TCMs.

### 2.4. Effect of Anticoagulation

Coagulation-related indicators of TT, PT, and APTT were measured to provide a baseline for research into the effects on invigorating blood circulation. All the results of clotting time assays are shown in Table 3.

TT was used to reflect the blood coagulation status. The transformation of fibrinogen into fibrin by interaction with thrombin and the prolongation of TT may indicate the inhibition of thrombin activity or fibrin polymerization [24]. As Table 3 shows, the anticoagulation activity in TT decreases significantly in CF, CF-GL, CF-GR, CF-AS, and CF-SL samples knocked out by HSYA and the order is CF-GL > CF-GR > CF > CF-SL > CF-AS. So the results illustrated that HSYA had the greatest effect in terms of extending TT in these herb pairs. Similarly, ASYB played the same role in CF-PS, CF-GL, CF-AS, and CF-SL samples and the order of the prolongation of TT is CF-GL > CF-SL > CF-PS > CF-AS. The corresponding active role of the two components had failed to show when safflower was combined with the other TCMs in addition to the above compatibility in this study.

PT was used to characterize the extrinsic coagulation factors and prolongation of PT indicated the inhibition of the extrinsic part of coagulation [19]. As shown in Table 3, prolongation of PT remarkably reduces after knocking out the HSYA in CF-PS, CF-GL, CF-GR, CF-AS, CF-SL, CF-AR, CF samples; the order of PT prolongation effect contribution degree is CF-SL > CF-AR > CF-AS > CF-GL > CF-GR > CF-PS > CF. ASYB played the same role in CF-PS, CF-GL, CF-SM, CF-AS, CF-SL, CF-AR, CF samples and the order is CF-SL > CF-AS > CF-GL > CF-PS > CF-SM > CF > CF-AR. Therefore, the results illustrated that the two compounds were indispensable components of safflower and the above series of herb pairs having a prolongation effect on PT and the corresponding active role of the two components had failed to show in the other samples.

Prolongation of APTT indicated an inhibition of the intrinsic and/or common system, because APTT was often used for the evaluation of coagulation factors in the intrinsic blood coagulation pathway [13]. In safflower and its series of herb pairs, prolonging APTT activity was significantly shortened after knocking out the HSYA or ASYB, as Table 3 shows. So the results revealed that HSYA had the greatest effect and ASYB was an indispensable component of APTT prolongation, respectively. The order of contribution degree was as follows: CF-SM > CF-AR > CF-SL > CF-GL > CF-GR > CF-PS > CF > CF-AS (knock-out HSYA) and CF-AR > CF-SM > CF-SL > CF-PS > CF-GL > CF > CF-AS > CF-GR (knock-out ASYB).

### 2.5. Integration of Activating Blood Circulation Effect with Multi-Index Comprehensive Index Method

**Figure 18:**
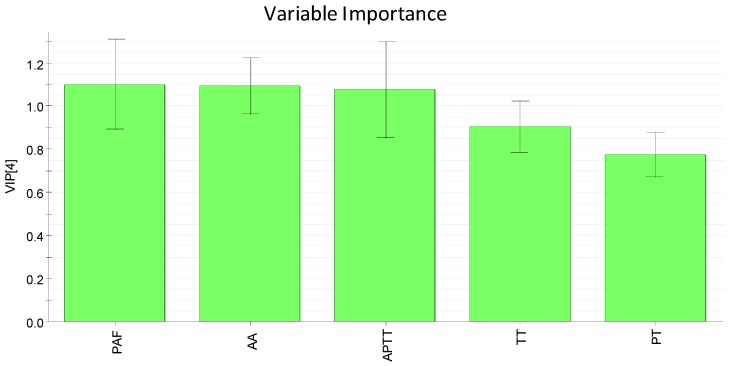
The VIP plot of activating blood circulation indexes of safflower series of herb pairs.

**Figure 19:**
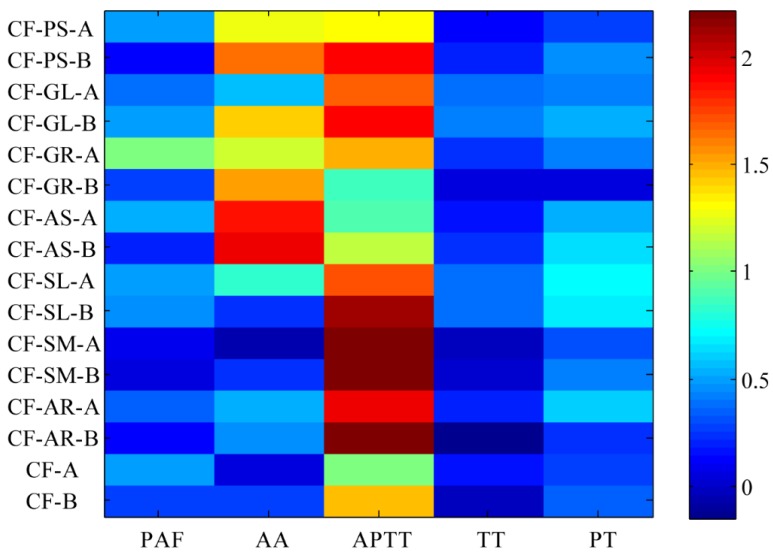
Hot map of contribution to different biological activities in the safflower series of herb pairs.

The source data of all the indexes of the samples were analyzed with EZ info in MassLynx v4.1 software (Waters Corporation, Milford, MA, USA) and Variable Importance (VIP) values were obtained by the partial least squares analysis; the greater the VIP value was, the greater contribution the variable made to promoting blood circulation and removing blood stasis with VIP > 1 as the significant variable [20]. The result was that the VIP values of PAF, AA, and APTT were all 1.1; TT and PT were 0.9 and 0.8, respectively, as shown in Figure 4. Because the VIP values of PAF, AA, and APTT were greater than 1 while those of TT and PT were less than 1, each target was given a weight coefficient: PAF, AA and APTT take a weight coefficient of 2, while TT and PT take a weight coefficient of 1. Based on comprehensively considering the integration results and the contributions of the compositions of different indicators (Table 4, Figure 18), both HSYA and ASYB made a certain contribution to activating blood circulation in the safflower and its series of herb pairs. A heat map (Figure 19) indicates that most of the samples had an obvious effect on AA-induced platelet aggregation and APTT. On the whole, the contribution of HSYA was bigger in CF-GR and CF-SL; the second was CF-AS, CF-AR, CF-PS, and CF-GL; function is relatively weak in CF and CF-SM, as follows: CF-GR > CF-SL > CF-AS > CF-AR > CF-PS > CF-GL > CF-SM > CF. The degree of contribution of ASYB in all samples was CF-GL > CF-PS > CF-AS > CF-SL > CF-SM > CF-AR > CF-GR > CF.

According to the above study and the results analysis, HSYA and ASYB made a contribution to the total bio-activity reflection of safflower and its series of herb pairs directly and indirectly. Simultaneously, the differences of the bioactivities of HSYA and ASYB in safflower and its series of herb pairs were not obvious as a whole. However, the differences were apparent under the conditions of different compatibilities, e.g., the contribution of HSYA was stronger than ASYB in the CF-GR herb pair, contrary to CF-GL.

## 3. Materials and Methods

### 3.1. Plant Materials

CF (the flower of *Carthamus tinctorius* L.) from Yili County, Xinjiang Province, China and PS (the mature seed of *Prunus persica* (L.) Batsch or *Prunus davidiana* (Carr.) Franch.) from Xia County, Shanxi Province, China were collected in July 2015. AS (the radix of *Angelica sinensis* (Oliv.) Diels) from Min County, Gansu Province, China, AR (the radix of *Scutellaria baicalensis* Georgi) from Wuchuan County, Neimenggu Province, China, SM (the radix and rhizoma of *Saliva miltiorrhiza* Bge.) from Zhongjiang County, Sichuan Province, China, SL (the heartwood of *Caesalpinia sappan* L.) from Tianyang County, Guangxi Province, China and GL (the radix and rhizoma of *Glycyrrhiza uralensis* Fisch., *Glycyrrhiza inflata* Bat. Or *Glycyrrhiza glabra* L.) from Yanchi County, Ningxia Province, China were collected in October 2015. GR (the steamed radix and rhizoma of the cultivar of *Panax ginseng* C. A. Mey.) was collected at Fusong County, Jilin Province, China in September 2015. They were identified morphologically by Dr. Hui Yan of the Department of Medicinal Plants, Nanjing University of Chinese Medicine, Nanjing, China. The voucher specimens (No. NJUTCM-20151109–20151116) were deposited in the Herbarium of the Nanjing University of Chinese Medicine.

### 3.2. Instrumentation

Prep-HPLC was performed using a Waters AutoPurification^TM^ system (Waters Corporation, Milford, MA, USA) equipped with a 2545 Binary High Voltage Gradient Pump, a 2767 Automatic Sampler and a 2489 Double Wavelength Detector (Waters Corporation, Milford, MA, USA), and Fractionlynx^TM^ software (Waters Corporation, Milford, MA, USA). XBridge^TM^ prep C_18_ OBD^TM^ column (30 mm × 150 mm, 5 μm, Waters Corporation, Milford, MA, USA) was applied for all experiments of the sample preparation. Chromatographic analysis was performed on a Waters HPLC system (Waters Corporation) equipped with a 2695 Separations Module and a 2998 Photodiode Array Detector. The analytical column employed was a Hypersil ODS2 column (4.6 mm × 250 mm, 5 μm, Thermo Corporation, Waltham, MA, USA). Finally, bioactive experiments were performed on a semi-automated coagulation analyzer named LG-PABER-1 (Steellex, Beijing, China).

### 3.3. Chemicals and Reagents

HSYA (purity > 96.5%) was purchased from the National Institute for the Control of Pharmaceutical and Biological Products (20150827, Beijing, China). ASYB (purity > 98%, by HPLC) was previously isolated from *Carthamus tinctorius* L. in our laboratory, and identified by UV spectra, MS, ^1^H-NMR, and ^13^C-NMR (Supplementary Materials) [21,22].

Arachidonic acid (AA) and platelet activating factor (PAF) were purchased from Sigma-Aldrich Shanghai Trading Co. Ltd. (Shanghai, China). Thrombin, Tris-HCl, and kits for prothrombin time (PT) (ISI 1.0) and activated partial thromboplastin time (APTT) assay were commercial reagents from Steellex (Beijing, China). Methanol was HPLC-grade from Merck (Darmstadt, Germany), ultra-pure water was purified by an EPED super purification system (EPED Technology Development Co. Ltd., Nanjing, China), and other reagents were of analytical grade.

### 3.4. Animals

Male New Zealand long-eared rabbits (2.0–2.5 kg) were provided by Qinglongshan Laboratory Animal (Nanjing, China). Before the experiment began, the animals were kept in a well-ventilated room with free access to food and water at a temperature of 20 ± 2 °C and a relative humidity of 55% ± 5%, with a 12 h light–dark cycle for one week. All animal welfare and experimental procedures were strictly in accordance with the guidelines for the care and use of laboratory animals (National Research Council of USA, 1996) and the related ethical regulations of Nanjing University of Chinese Medicine.

### 3.5. Preparation of Herb Pair Samples

CF and mixed crude herbs of CF and PS, SM, AS, SL, AR, GR, and GL, respectively, at the ratio of 1:1 (*w*/*w*) were weighed out in 100 g each, and then extracted twice in 10 or 8 times the quantity of water, with a heating reflux time of 2 and 1.5 h. The extracting solution was combined and the solvent was concentrated below 50 °C till the ratio of 1:1 (*w*/*v*, 1 g of crude drugs and 1 mL of the extracted filtrates) under a vacuum to obtain CF and herb pair (CF-GL, CF-AR, CF-AS, CF-SL, CF-PS, CF-GR, CF-SM) samples.

### 3.6. Preparation of Safflower Series of Herb Pairs Knock Out HSYA or ASYB

The right amount of samples was dissolved with 10% methanol in an ultrasonic machine and its concentration was almost 300 mg mL^−1^. Then the samples were centrifuged at 13,000 rpm for 10 min before injection. The injection volume was 500 μL; the mobile phase consisted of water (A)/methanol (B) at a flow rate of 30 mL/min. We used a gradient elution for preparative HPLC, which was programmed as follows: condition I: 0 min–10% B; 5 min–15% B; 15 min–20% B; 18 min–100% B; 21 min–100% B; 23 min–10% B and 28 min–10% B (for HSYA), condition II: 0 min–10% B; 5 min–20% B; 20 min–25% B; 22 min–100% B; 25 min–100% B; 27 min–10% B and 29 min–10% B (for ASYB); the detection wavelength was set at 270 nm (stronger absorption wavelength of all the ingredients) or 400 nm (λ_max_). The target component was collected according to the retention time of HSYA and ASYB with 10 mL per tube. The remnant solutions were collected in a 5-L flask, and then the solvent was recycled at 50 °C under a vacuum. So all samples, including HSYA, ASYB, and safflower series of herb pairs achieved knock-out of HSYA or ASYB after freeze-drying.

### 3.7. Evaluation of the Antiplatelet Aggregation and Anticoagulation Activities in Vitro

#### 3.7.1. Sample Preparation

Before and after knock-out, samples were weighed accurately and dissolved in deionized water to obtain sample solutions of the concentration of 0.4 g crude drugs/g or 0.8 g crude drugs/g. Afterwards the former was used to measure the activated partial thromboplastin time (APTT) and the latter was provided to measure the thrombin time (TT), prothrombin time (PT), the platelet aggregation induced by arachidonic acid (AA), and the platelet activating factor (PAF).

#### 3.7.2. Detection of Antiplatelet Aggregation

Male New Zealand long-eared white rabbits were allowed to fast for 8 h with free access to water prior to the experiment. Blood was collected through a polyethylene cannula placed in the common carotid artery and stored in a 10 mL plastic flask containing 3.8% sodium citrate (*v*/*v*, 1:9) after 30 mg·kg^−1^ 3% pentobarbital sodium anesthesia, then centrifuged at 800 rpm for 10 min to obtain the upper platelet-rich plasma (PRP) and further centrifuged at 3000 rpm for 10 min to prepare platelet-poor plasma (PPP) [23,24].

Furthermore, stirring beads, 10 μL testing drug, and 280 μL PRP were placed together in the test cup, and 10 μL sample solvent and 280 μL PRP were mixed together to form a control group, all incubated at 37 °C for 3 min. To the samples in the test channel were added 10 μL inducer (PAF or AA), then the maximum aggregation was determined within 6 minutes with the PPP set at zero at any time; we further calculated the platelet aggregation inhibition rate according to the following formula: platelet aggregation inhibition rate (%) = (maximum aggregation rate in the placebo group − the medicated group) / maximum aggregation rate × 100.

#### 3.7.3. Determination of Blood Coagulation Function (TT, PT, APTT)

The anticoagulation activity was measured by clotting assay of TT, PT, and APTT according to the methods provided by the biological reagents provider [13]. Briefly, TT was measured by incubating a 10 μL sample with 50 μL PPP for 3 min at 37 °C, then adding 50 μL thrombin (10 U mL^−1^) that had been diluted with Tris-HCl buffer solution (pH 7.4); the clotting time extension rate was calculated after recording the blood coagulation time according to the following formula: clotting time extension rate (%) = (blood coagulation time in the medicated group − the placebo group) / blood coagulation time in the placebo group × 100.

PT was measured by incubating a 10 μL sample with 50 μL PPP for 3 min at 37 °C, then the clotting time was recorded after adding 100 μL PT agent and the computational formula was in accordance with TT.

APTT was measured by incubating a 10 μL sample and 50 μL PPP with 50 μL APTT agent for 3 min at 37 °C, then the clotting time was recorded. This was followed by adding 100 μL of warmed 20 mM CaCl_2_ and the computational formula was in accordance with TT.

#### 3.7.4. Statistical Analysis

Antiplatelet aggregation and anticoagulation tests of each sample were conducted four times in four channels. All data were presented as mean ± SD. The differences were carried out by Student’s *t*-test with *p* < 0.05 to assess the statistical significance of the relationship between the tested samples and the control [13].

In addition, the platelet aggregation, TT, PT, and APTT multiple indicators were simply processed with MassLynx v4.1 (Waters) analysis to obtain the Variable Importance (VIP) value of each indicator. On the basis of the VIP value, each index was given a weight coefficient. Subsequently, all data must be standardized as the difference between the values before and after knocking out HSYA or ASYB divided by the original samples when the corresponding index value of the samples’ knocked-out target ingredients increases compared to the original samples. A heat map of the standardized values of each sample for all the bioactivity indexes calculated above was generated, allowing direct comparison of the varied bioactivities of the different samples on the five indexes. Finally, the total contribution of activating blood circulation was calculated for safflower series of samples using a multi-index comprehensive index method [25].

## 4. Conclusions

For a long time, it has been widely accepted in TCM theory that herb pairings are responsible for a drug’s biological activities [7,13,26]. So it is very difficult to illuminate the actual effect of a single herb and its bioactive components within an herb pair. In this study, we successfully utilized a convenient and effective approach to selectively knock out some bioactive constituents of TCMs with prep-HPLC followed by biological activity evaluation. Using the method, we could further define bioactive components based on biological activity differences before and after knocking out target compounds and compare their contribution to the whole herb pair. So, this method may achieve hierarchical identification of bioactive components in a medicinal herb.

Our results also indicated that the effect of safflower series of herb pairs in activating blood circulation and removing stasis was stronger than for a single herb and the bioactive ingredients played different roles in these herb pairs. Furthermore, the different contribution of HSYA and ASYB in different herb pairs suggested a variation in the bioactive components for herb-pair compatibilities. The study gave a meaningful demonstration of the suitability of TCM to reveal bioactive compounds and their roles. Though bioavailability and metabolism were not considered using the in vitro approach in our study, it still provided a scientific basis for elucidating the compatibility mechanism of herb pairs, paving the way for clinical applications and new drug development using the safflower series of herb pairs.

## Figures and Tables

**Table 1:** Samples before and after knocking out the target component and knock-out constituents in safflower series of herb pairs on PAF-induced platelet aggregation (mean ± SD, *n* = 4).

Group	Aggregation Ratio (%)	Aggregation Inhibition Ratio (%)	Contribution Degree (%)
Negative control	85.83 ± 4.70	—	—
Positive control	45.65 ± 1.78 **	46.81 ± 5.33	—
HSYA	72.40 ± 7.18 *	15.64 ± 8.37	—
ASYB	54.35 ± 12.31 **	36.67 ± 14.34	—
CF	33.08 ± 3.43 **	61.46 ± 4.00	—
CF-A	46.48 ± 6.66 **^△^	45.85 ± 7.76	25.40
CF-B	40.55 ± 3.52 **^△^	52.75 ± 4.10	14.17
CF-PS	51.00 ± 4.07 **	40.58 ± 4.74	—
CF-PS-A	59.45 ± 5.53 **^△^	30.73 ± 6.44	24.26
CF-PS-B	53.47 ± 3.35 **	37.70 ± 3.91	—
CF-GL	46.83 ± 3.68 **	45.44 ± 4.28	—
CF-GL-A	54.30 ± 5.28 **	36.73 ± 6.15	—
CF-GL-B	56.25 ± 7.50 **	34.46 ± 8.74	—
CF-GR	18.28 ± 4.04 **	78.71 ± 4.71	—
CF-GR-A	53.18 ± 10.34 **^△△^	38.04 ± 12.05	51.67
CF-GR-B	27.25 ± 5.57 **^△^	68.25 ± 6.49	13.29
CF-AS	11.95 ± 5.57 **	86.08 ± 2.58	—
CF-AS-A	31.08 ± 2.65 **^△△^	63.79 ± 3.06	25.89
CF-AS-B	18.73 ± 4.82 **^△^	78.18 ± 5.62	9.17
CF-SL	36.45 ± 4.42 **	57.53 ± 5.16	—
CF-SL-A	48.78 ± 5.72 **^△^	43.17 ± 6.66	24.96
CF-SL-B	47.53 ± 6.17 **^△^	44.63 ± 7.19	22.43
CF-SM	25.60 ± 6.53 **	70.17 ± 7.61	—
CF-SM-A	28.45 ± 6.27 **	66.85 ± 7.31	—
CF-SM-B	27.03 ± 4.35 **	68.51 ± 5.07	—
CF-AR	28.98 ± 5.71 **	66.24 ± 6.65	—
CF-AR-A	38.53 ± 3.70 **^△^	55.11 ± 4.31	16.80
CF-AR-B	32.65 ± 6.06 **	61.96 ± 7.06	—

* *p* < 0.05, ** *p* < 0.01 vs. control group. ^Δ^ *p* < 0.05, ^ΔΔ^ *p* < 0.01 vs. the respective original solutions. Positive control was ginkgolide B at the concentration of 0.5 mg·kg^−1^ for PAF induced platelet aggregation assay. The sample concentration was 0.8 g crude drugs/g and the monomer components (HSYA, ASYB) were basically the same as in the original solutions.

**Table 2:** Samples before and after knocking out the target component and knock-out constituents in safflower series of herb pairs on AA-induced platelet aggregation (mean ± SD, *n* = 4).

Group	Aggregation Ratio (%)	Aggregation Inhibition Ratio (%)	Contribution Degree (%)
Negative control	78.38 ± 8.66	—	—
Positive control	58.23 ± 5.45 **	25.71 ± 3.44	—
HSYA	71.25 ± 2.12	9.09 ± 2.71	—
ASYB	73.15 ± 2.44	6.67 ± 3.11	—
CF	11.15 ± 2.14 **	85.77 ± 2.73	—
CF-A	12.78 ± 2.86 **^△^	83.70 ± 3.65	4.89
CF-B	20.25 ± 2.23 **^△△^	74.16 ± 2.85	9.43
CF-PS	11.04 ± 3.25 **	55.02 ± 14.09	—
CF-PS-A	63.07 ± 7.75 *^△△^	19.53 ± 2.70	64.50
CF-PS-B	70.75 ± 2.43 ^△△^	9.73 ± 3.10	82.32
CF-GL	14.78 ± 7.52 **	81.15 ± 9.60	—
CF-GL-A	32.45 ± 5.55 **^△△^	58.60 ± 7.08	27.79
CF-GL-B	59.60 ± 2.61 **^△△^	23.96 ± 3.33	70.48
CF-GR	42.22 ± 2.72 **	30.24 ± 4.50	—
CF-GR-A	53.18 ± 6.10 ^△^	12.14 ± 10.08	59.84
CF-GR-B	56.28 ± 3.20 ^△△^	7.02 ± 5.28	76.78
CF-AS	37.82 ± 2.85 **	37.50 ± 4.71	—
CF-AS-A	59.22 ± 5.00 ^△△^	2.15 ± 8.26	94.27
CF-AS-B	59.82 ± 6.66 ^△△^	1.16 ± 11.00	96.92
CF-SL	51.67 ± 0.91 **	34.08 ± 1.16	—
CF-SL-A	62.87 ± 2.48 *^△△^	19.79 ± 3.17	41.93
CF-SL-B	54.77 ± 2.50 **	30.12 ± 3.19	—
CF-SM	28.30 ± 3.35 **	63.89 ± 4.28	—
CF-SM-A	26.83 ± 3.61 **	65.77 ± 4.61	—
CF-SM-B	33.93 ± 4.14 **	56.71 ± 5.29	—
CF-AR	27.40 ± 7.36 **	65.04 ± 9.39	—
CF-AR-A	40.83 ± 4.40 **^△^	47.90 ± 5.62	26.35
CF-AR-B	39.22 ± 3.55 **^△^	50.41 ± 4.49	28.75

* *p* < 0.05, ** *p* < 0.01 vs. control group. ^Δ^ *p* < 0.05, ^ΔΔ^ *p* < 0.01 vs. the respective original solutions. Positive control was ginkgolide B at the concentration of 0.5 mg·kg^−1^ for AA induced platelet aggregation assay. The sample concentration was 0.8 g crude drugs/g and the monomer components (HSYA, ASYB) were basically the same as in the original solutions.

**Table 3:** Samples before and after knocking out the target component and knock-out constituents in safflower series of herb pairs on TT, PT, and APTT (mean ± SD, *n* = 4).

Group	TT		PT		APTT	
	t (s)	Contribution Degree (%)	t (s)	Contribution Degree (%)	t (s)	Contribution Degree (%)
Negative control	13.60 ± 0.50	—	9.65 ± 0.24	—	14.28 ± 0.22	—
Positive control	15.56 ± 1.22 **	—	14.08 ± 1.48 **	—	16.38 ± 0.54 **	
HSYA	15.15 ± 1.24	—	9.70 ± 0.24	—	14.13 ± 0.59	—
ASYB	13.75 ± 0.38	—	9.38 ± 0.70	—	14.25 ± 0.29	—
CF	45.30 ± 2.02 **	—	34.93 ± 2.19 **	—	71.40 ± 3.27 **	—
CF-A	40.05 ± 2.21 **	16.56	28.35 ± 0.88 **^△△^	26.03	42.68 ± 0.61 **^△△^	50.28
CF-B	45.83 ± 1.51 **	—	26.28 ± 1.78 **^△△^	34.22	29.90 ± 3.47 **^△△^	72.65
CF-PS	30.78 ± 1.01 **	—	18.23 ± 0.15 **	—	32.15 ± 3.45 **	—
CF-PS-A	28.40 ± 1.76 **	—	15.80 ± 0.26 **^△△^	28.32	20.30 ± 1.02 **^△△^	66.31
CF-PS-B	27.48 ± 2.50 **^△^	19.21	14.18 ± 0.15 **^△△^	47.20	15.20 ± 0.18 **^△△^	94.85
CF-GL	122.88 ± 27.26 **	—	153.7 ± 2.08 **	—	70.18 ± 9.82 **	—
CF-GL-A	81.13 ± 7.03 **^△△^	38.20	92.43 ± 4.72 **^△△^	42.53	22.68 ± 0.67 **^△△^	84.97
CF-GL-B	77.65 ± 3.64 **^△△^	41.39	77.40 ± 1.81 **^△△^	52.97	17.40 ± 1.92^*^^△△^	94.42
CF-GR	37.60 ± 0.96 **	—	25.53 ± 0.79 **	—	71.90 ± 4.58 **	—
CF-GR-A	32.05 ± 2.16 **^△△^	23.13	18.83 ± 1.05 **^△△^	42.19	28.80 ± 1.25 **^△△^	74.80
CF-GR-B	36.43 ± 4.02 **	—	24.95 ± 3.75 **	—	47.25 ± 5.96 **^△△^	42.78
CF-AS	52.80 ± 3.98 **	—	112.38 ± 14.81 **	—	97.38 ± 4.07 **	—
CF-AS-A	46.95 ± 0.48 **^△^	11.08	57.90 ± 1.93 **^△△^	53.03	59.85 ± 4.85 **^△△^	45.16
CF-AS-B	43.05 ± 4.11 **^△^	18.46	44.93 ± 0.52 **^△△^	65.66	49.70 ± 1.80 **^△△^	57.38
CF-SL	22.20 ± 0.71 **	—	15.40 ± 0.73 **	—	24.53 ± 2.68 **	—
CF-SL-A	18.98 ± 0.46 **^△△^	14.92	11.25 ± 0.26 **^△△^	72.77	15.70 ± 0.70 **^△△^	86.15
CF-SL-B	18.80 ± 0.29 **^△△^	24.87	11.40 ± 0.20 **^△△^	69.57	13.60 ± 0.39^*^^△△^	106.6
CF-SM	64.68 ± 14.47 **	—	62.57 ± 8.88 **	—	26.70 ± 2.73 **	—
CF-SM-A	65.08 ± 6.96 **	—	47.00 ± 4.53 **	—	12.91 ± 0.67 **^△△^	111.0
CF-SM-B	64.60 ± 9.01 **	—	41.10 ± 1.41 **^△^	40.57	13.10 ± 2.36^△△^	109.5
CF-AR	29.35 ± 1.52 **	—	19.68 ± 0.52 **	—	24.45 ± 2.59 **	—
CF-AR-A	25.90 ± 1.52 **	—	13.75 ± 0.13 **^△△^	59.12	14.53 ± 0.69^△△^	97.54
CF-AR-B	31.73 ± 1.50 **	—	17.28 ± 0.36 **^△△^	23.97	13.30 ± 0.67^*^^△△^	109.6

* *p* < 0.05, ** *p* < 0.01 vs. control group. ^Δ^ *p* < 0.05, ^ΔΔ^ *p* < 0.01 vs. the respective original solutions. Positive control was rivaroxaban at the concentration of 2.5 mg·kg^−1^. The sample concentration was 0.8 g crude drugs/g for TT and PT, 0.4 g crude drugs/g for APTT and the monomer components (HSYA, ASYB) were basically the same as in the original solutions.

**Table 4:** Total effect changes of activating blood in safflower series of herb pairs.

Group	Vsa			Vsb		Total Contribution Value of Activating Blood (2a + b)
	PAF	AA	APTT	TT	PT	
CF-PS-A	0.2427	0.6450	0.6631	0.1385	0.2832	3.5235
CF-PS-B	0.0710	0.8232	0.9485	0.1921	0.4720	4.3494
CF-GL-A	0.1917	0.2779	0.8497	0.3820	0.4253	3.4460
CF-GL-B	0.2416	0.7047	0.9442	0.4139	0.5297	4.7247
CF-GR-A	0.5167	0.5985	0.7480	0.2313	0.4219	4.3797
CF-GR-B	0.1329	0.7679	0.4278	0.0488	0.0365	2.7424
CF-AS-A	0.2589	0.9427	0.4516	0.1492	0.5303	3.9860
CF-AS-B	0.0918	0.9691	0.5738	0.2487	0.6566	4.1745
CF-SL-A	0.2496	0.4193	0.8615	0.3744	0.7217	4.1569
CF-SL-B	0.2242	0.1162	1.0663	0.3953	0.6957	3.9045
CF-SM-A	0.0473	−0.0294	1.1103	−0.0078	0.2942	2.5428
CF-SM-B	0.0237	0.1124	1.0950	0.0016	0.4057	2.8694
CF-AR-A	0.1680	0.2635	0.9754	0.2190	0.5912	3.6242
CF-AR-B	0.0646	0.2249	1.0964	−0.1511	0.2393	2.8600
CF-A	0.2540	0.0241	0.5028	0.1656	0.2603	1.9877
CF-B	0.1417	0.1354	0.7265	−0.0167	0.3422	2.3327

## Supplementary Materials

The following are available online at http://www.mdpi.com/1420-3049/21/11/1480/s1.
