# Supplementary material for: Comparative Analysis of the Effects of Hydroxysafflor Yellow A and Anhydrosafflor Yellow B in Safflower Series of Herb Pairs Using Prep-HPLC and a Selective Knock-Out Approach
Source: Molecules. 2016 Nov 6;21(11):1480. doi: 10.3390/molecules21111480 (PMC6274300; doi:10.3390/molecules21111480)
Supplement: Supplementary file 1 [file molecules-21-01480-s001.pdf]

Supplementary Materials: Comparative Analysis of Effects of Hydroxysafflor Yellow A and Anhydrosafflor Yellow B in Safflower Series of Herb Pairs by Prep-HPLC and a Selective Knock-Out Approach

Cheng Qu, Lin-Yan Wang, Wen-Tao Jin, Yu-Ping Tang, Yi Jin, Xu-Qin Shi, Li-Li Shang, Er-Xin Shang and Jin-Ao Duan

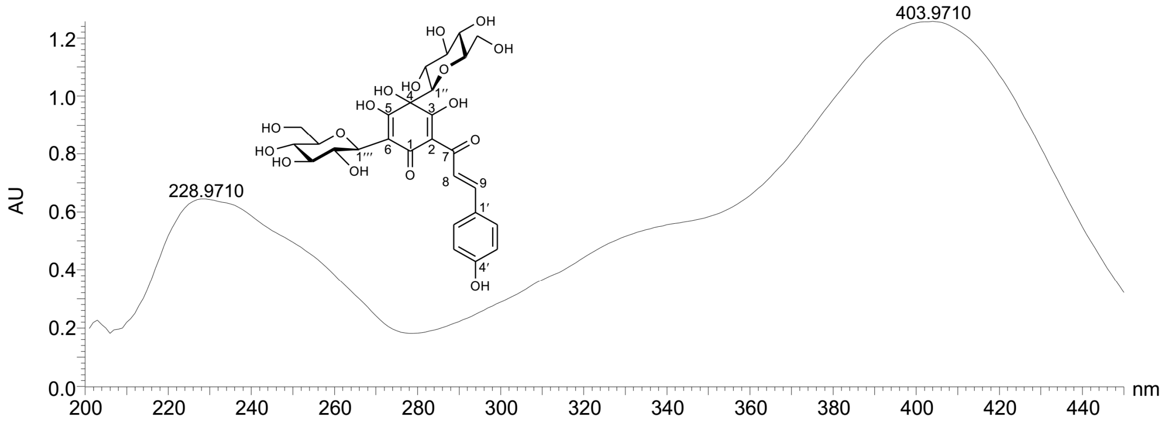

Figure S1. UV spectrum of HSYA.

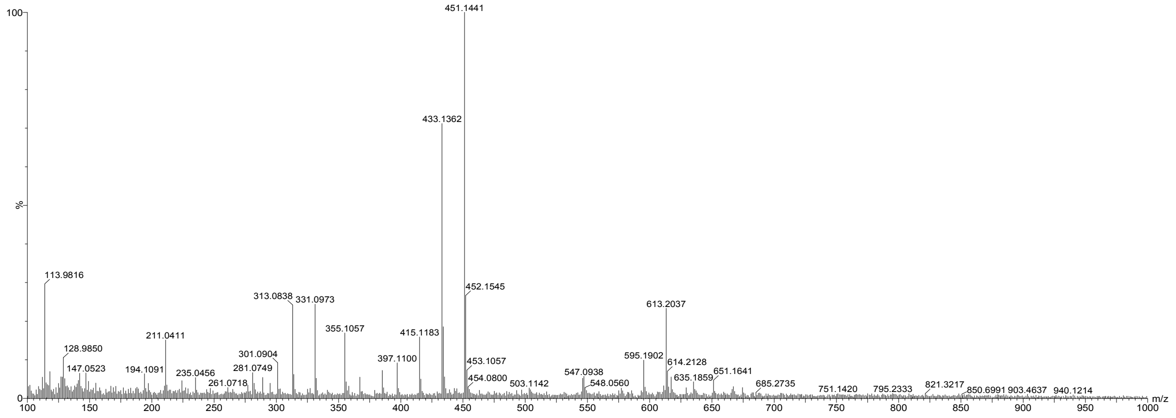

Figure S2. ESI$^+$ spectrum of HSYA.

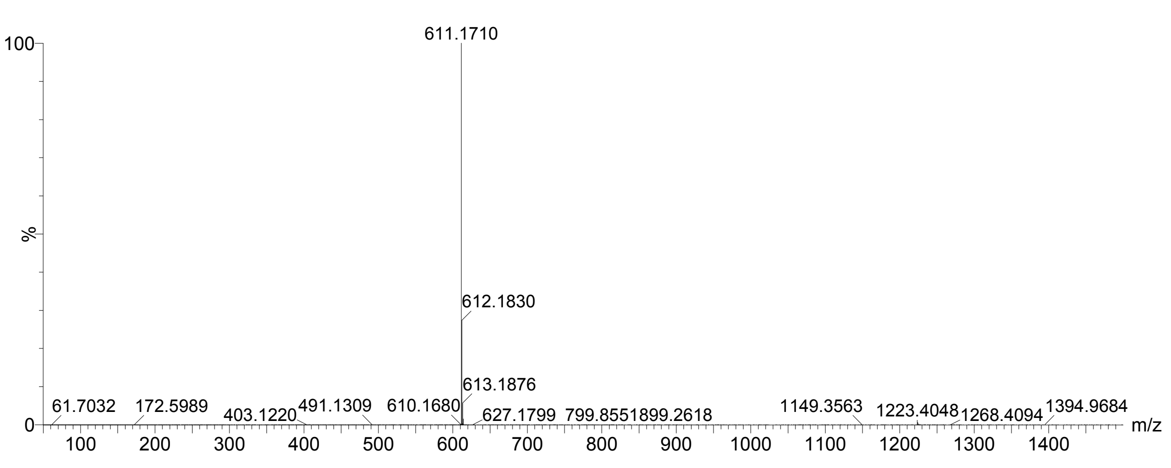

Figure S3. ESI$^-$ spectrum of HSYA.

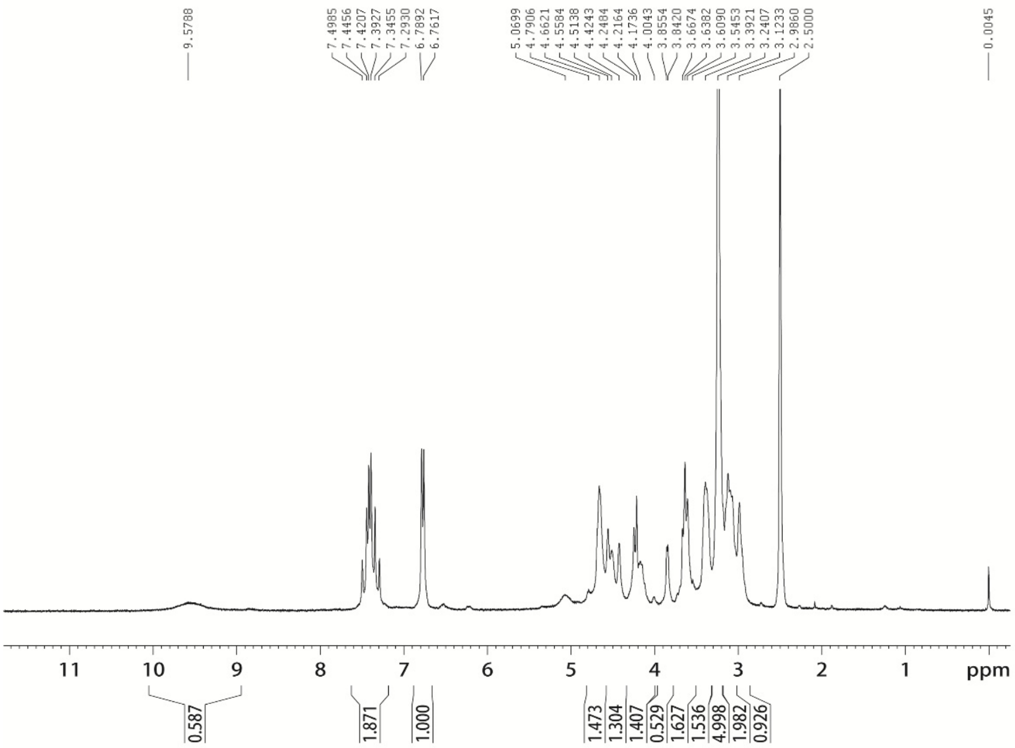

Figure S4. ^{1}H-NMR spectrum of HSYA in DMSO-d_6 (300 MHz).

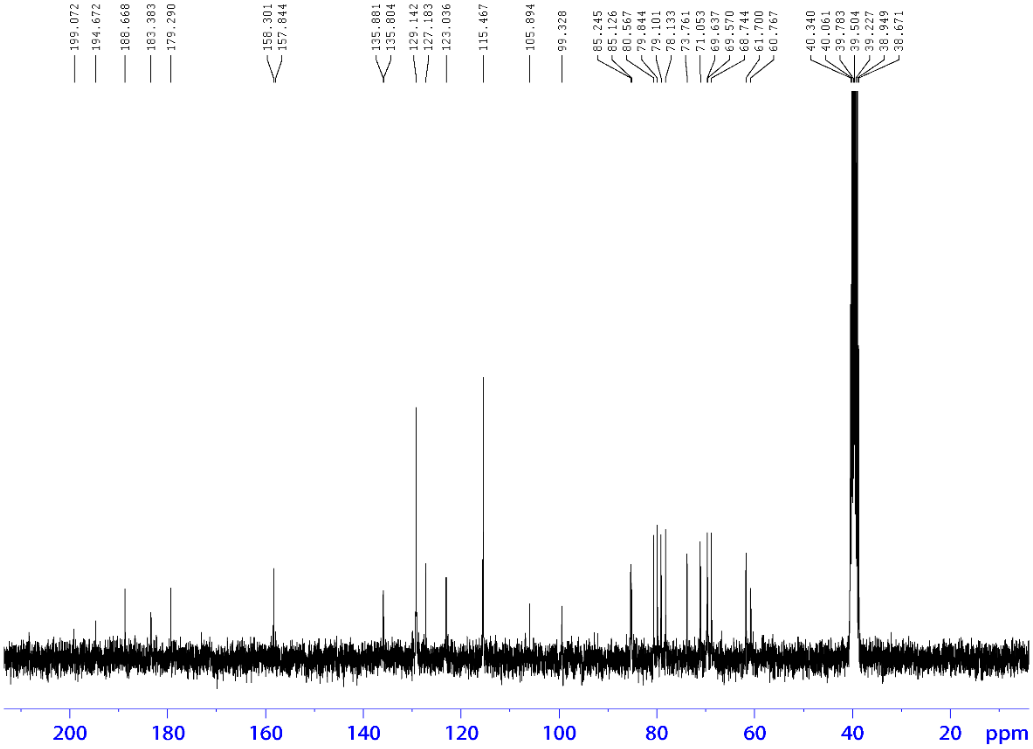

Figure S5. ^{13}C-NMR spectrum of HSYA in DMSO-d_6 (75 MHz).

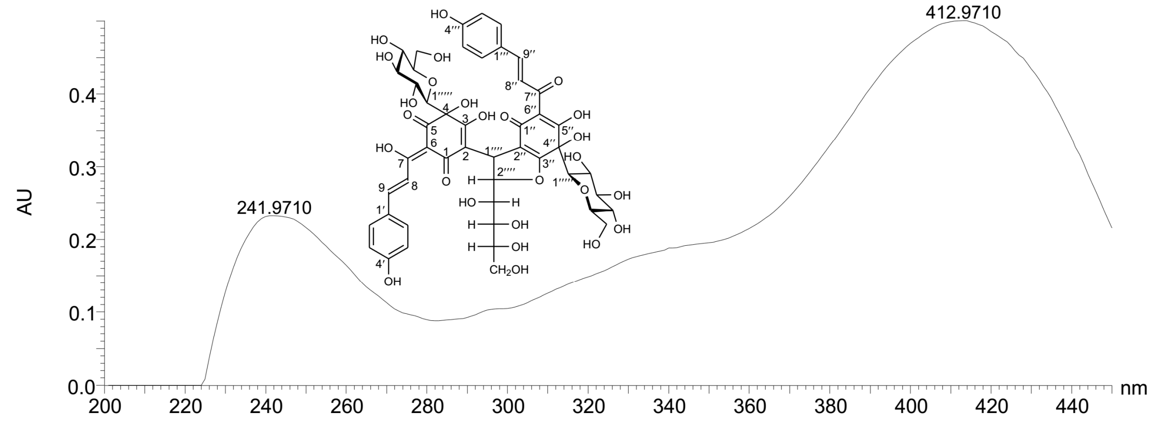

Figure S6. UV spectrum of ASYB.

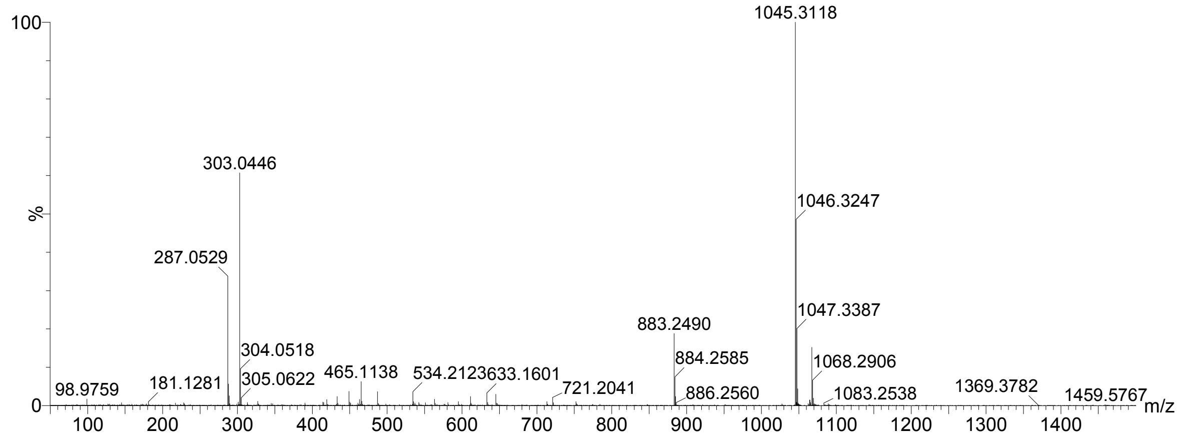

Figure S7. ESI$^+$ spectrum of ASYB.

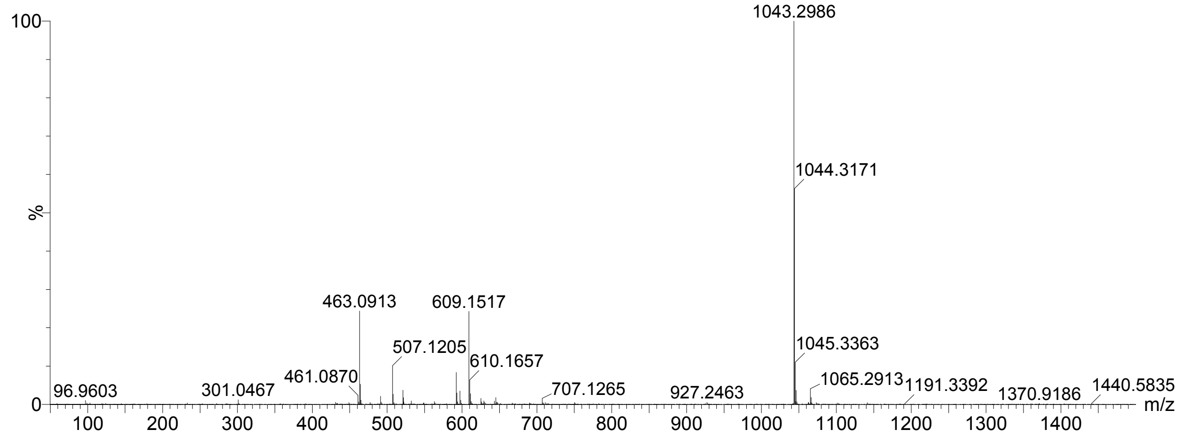

Figure S8. ESI$^-$ spectrum of ASYB.

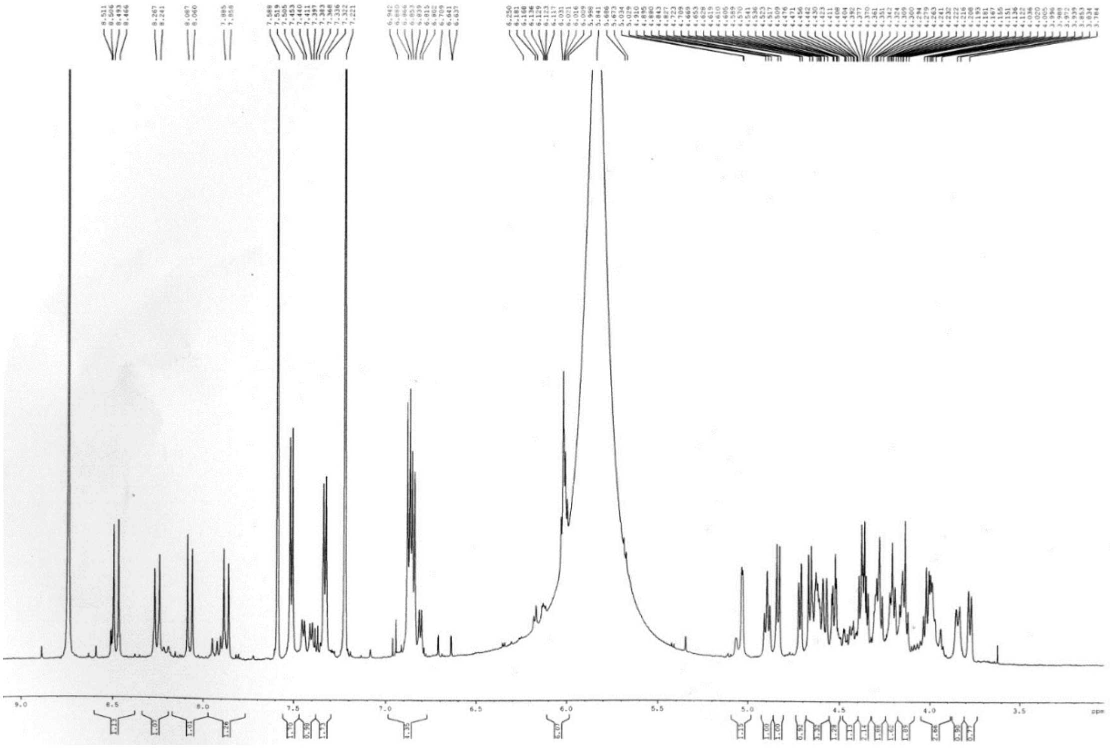

Figure S9. ^{1}H-NMR spectrum of ASYB in DMSO-d_6 (600 MHz).

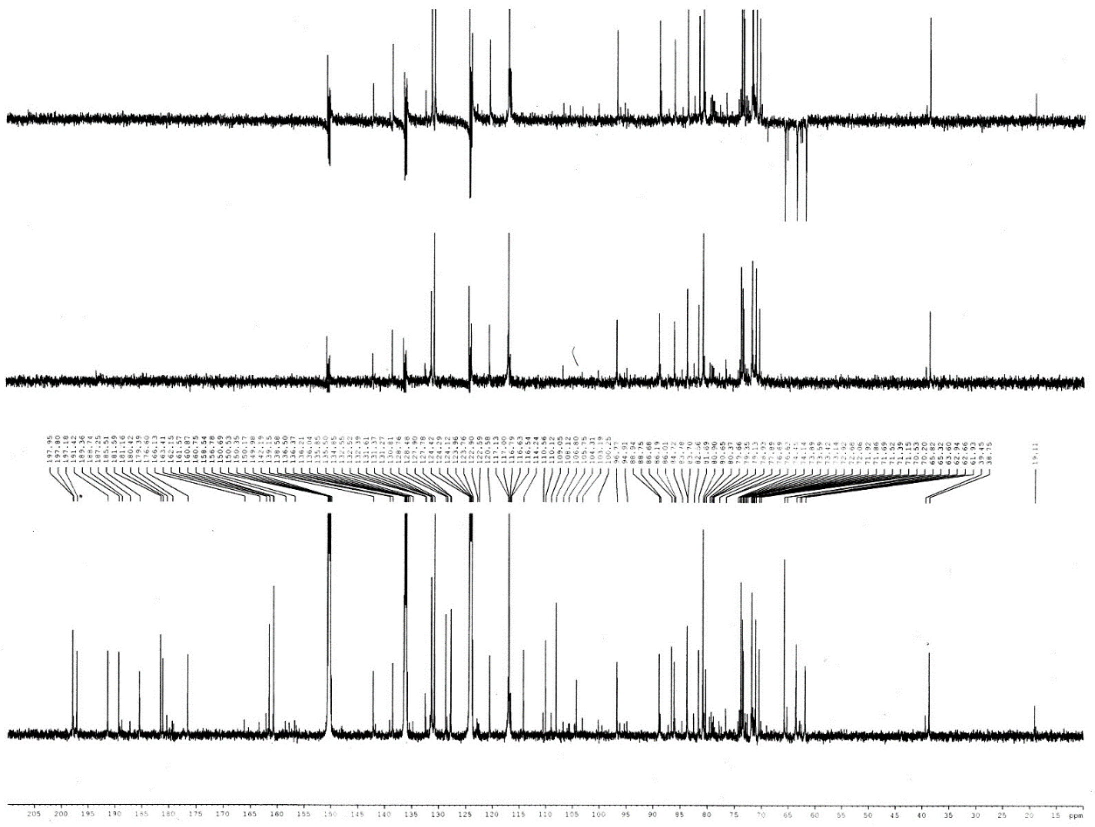

Figure S10. ^{13}C-NMR spectrum of ASYB in DMSO-d_6 (150 MHz).
